# Research highlights, *The Plant Genome*, Volume 13, Issue 2

**DOI:** 10.1002/tpg2.20042

**Published:** 2020-07-01

## 1 WHEN NEMATODES ATTACK

Plant parasitic nematodes are the bane of potato growers around the world because there are so few effective ways to fight them. However, some wild plants, such as *Solanum sisymbriifolium* (SSI), are able to halt the life‐cycle of invading cyst nematodes long before the animals become sexually mature. Wixom et al. (https://doi.org/10.1002/tpg2.20016) have identified 277 genes that change expression shortly after this plant's roots are invaded by these nematodes. Sixty percent of these changes did not occur when plants were treated with generic mediators of pathogen responses such as salicylic acid, methyl jasmonate, or wounding. It is possible that among these genes that only respond to nematodes are components of the unique resistance of SSI to cyst‐forming nematodes.

## 2 FOUR GENES MAKE ORNAMENTAL TOMATO

Tomato can be used as an ornamental plant and a vegetable crop. There are few genetic studies of ornamental traits for the development of ornamental tomato varieties. Safaei et al. (https://doi.org/10.1002/tpg2.20017) identified four genetic loci for ornamental traits by linkage mapping. SNP markers tightly linked to the loci were developed to select compact tomato plants with yellow pear‐shaped fruits. This approach can accelerate breeding programs for ornamental tomatoes.

## 3 M6A METHYLATION INVOLVED IN THE CALLUS INDUCTION

Callus induction is the process of pluripotent stem cell formation in plants, which requires precise epigenetic regulation. Du et al. (https://doi.org/10.1002/tpg2.20018) reported that the m6A methylation, an important post‐transcriptional epigenetic modification, changed significantly during the callus induction of maize, especially at the time of callus initiation. At this time, some unique methylated genes were found, including some key genes for callus induction. Moreover, this unique methylation preferred to increase the expression level of these genes.

## 4 MANGROVE PLASTID GENOMES EVOLVE INDEPENDENTLY

Mangrove species evolved separately from terrestrial plants to convergently adapt to saline, anoxic habitat, and provide an ideal case for studying convergent evolution at the genomic level. Han et al. (https://doi.org/10.1002/tpg2.20019) demonstrate the commonness as well as uniqueness of plastid genomes between representative mangrove species and terrestrial relatives. Both genome structure and sequence variation were found conserved among phylogenetic related species other than among mangrove species from different lineage. The evolution of mangrove plastid genomes was more related to lineage‐specific history than convergent adaptation, and convergent evolutionary dynamics leaves no significant signal in the plastid genome of mangroves.

## 5 GENES RESPOND TO COLD STRESS IN RICE

Cold stress could adversely affect crop growth and productivity. Yue et al. (https://doi.org/10.1002/tpg2.20020) report that a stress‐activated protein kinase and a calcium‐dependent protein kinases significantly correlated with cold tolerance of rice. They are involved in the regulation of cold tolerance through the plant‐pathogen interaction pathway and plant hormone signal transduction pathway. Gene polymorphism can affect the cold tolerance of rice. It provides a molecular foundation for the breeding of cold‐resistant rice.

## 6 GENOME BAYESIAN LINEAR AND NON‐LINEAR REGRESSION FOR ORDINAL DATA

Linear and non‐linear models used in applications of genomic selection (GS) can fit different types of responses. Genomic‐enabled prediction models include linear, non‐linear and non‐parametric models, mostly for continuous responses and less frequently for categorical responses. Several linear and non‐linear models are special cases of a more general family of statistical models known as artificial neural networks. In this paper, Pérez‐Rodríguez et al. (https://doi.org/10.1002/tpg2.20021) propose a Bayesian Regularized Neural Network (BRNNO) scheme for modelling ordinal data. Results showed that the BRNNO model performed better in terms of genomic‐based prediction than the other models.

## 7 FERTILITY FACTORS PERTURB MAIZE MITOPROTEOME

The major players of life, proteins, are influenced by the sterile factor and the restorer gene within anther mitochondria in cytoplasmic male sterility maize. Zhang et al. (https://doi.org/10.1002/tpg2.20022) report that the unknown sterile gene hinders proteins related to energy metabolism, which confirmed the proposed energy deficiency hypothesis. Whereas the restorer gene meets the energy requirement by activating the alternative metabolic pathways and by improving the post‐translation processing efficiency of proteins in energy‐producing complexes to restore pollen fertility. The latter results bring whole new views on the restoration mechanism of the *Rf4* gene.

## 8 LIPASE PRECURSORS TO FIGHT *Fusarium* MYCOTOXINS

*Fusarium langsethiae* produces T‐2 and HT‐2 mycotoxins that are accumulated into oat grain and can cause higher toxicity to mammals. The most efficient way to reduce the concentration of these mycotoxins in grain is to develop new resistant varieties. Currently, there is no information on where the resistance genes can be located within the oat genome. Isidro et al. (https://doi.org/10.1002/tpg2.20023) found five single nucleotide polymorphisms in the linkage group Mr06 with T‐2 + HT‐2 mycotoxin accumulation. The marker *avgbs_6K_95238.1* mapped within genes showing similarity to lipase, lipase‐like or lipase precursor mRNA sequences and zinc‐finger proteins. These regions have previously been shown to confer a significant increase in resistance to *Fusarium* species.

## 9 MACHINE LEARNING ANALYSES OF METHYLATION PROFILES UNCOVERS TISSUE‐SPECIFIC GENE EXPRESSION PATTERNS IN WHEAT

DNA methylation is a dynamic regulator of the plant genome during development and environmental adaptions. Aberrant patterns of DNA methylation can lead to developmental abnormalities and in extreme biotic/abiotic stress conditions, it could simply mean the survival of the plant. N'Diaye et al. (https://doi.org/10.1002/tpg2.20027) showed a strong correlation (*p* value = 9.20 × 10^−10^) between methylation profile and gene expression. We identified tissue‐specific gene regulation patterns with high confidence (accuracy = 0.81).

## 10 DECIPHERING FERTILITY TRANSITION IN PIGEONPEA

Pigeonpea is an important pulse crop of the semi‐arid tropics. The discovery of a three‐line hybrid breeding system was successful in breaking the longstanding yield barrier. Further, a two‐line hybrid system was explored to simplify and enhance the efficiency of the hybrid breeding. Pazhamala et al. (https://doi.org/10.1002/tpg2.20028) report identification of a thermosensitive male sterile line (*Envs Sel107*) which precisely responds to day‐temperature for its use in a two‐line hybrid breeding system. Multiomics analyses along with cellular level studies of stage‐specific anthers revealed key candidate genes involved in auxin homeostasis, cell wall modification, and sugar transport, impairment of which could have led to male sterility. Thus, a molecular mechanism that underpins the fertility transition in response to day‐temperature was outlined.

## 11 CADMIUM TOXICITY DETERIORATES GRAIN MINERAL COMPOSITION

A high cadmium uptake from soil deteriorates grain mineral composition of crop plants and toxifies the grains, and, consequently, harms human health when contaminated grains are consumed. Safdar et al. (https://doi.org/10.1002/tpg2.20030) report five regions on the wheat genome that harbor genes controlling cadmium uptake to aerial plant parts. Their study further shows that variations in these genomic regions cause variation in cadmium uptake. Moreover, they report that based on cadmium uptake in leaves and its translocation to grains, various genotypes can be categorized into three groups: hyper‐accumulators, which accumulate high cadmium in leaves but the translocation to grains remains low; hyper‐translocators, which translocate most of the cadmium they uptake to grains and leaf cadmium content remain low; and moderate lines, in which the uptake of cadmium from soil remains low.

## 12 SIGNATURE OF SELECTION IN A SOFT WHEAT POPULATION

Artificial selection increases the frequency of favorable alleles. These changes in the allele frequencies can be tracked to identify genomic regions under selection that are valuable for breeding. Gaire et al. (https://doi.org/10.1002/tpg2.20031) identified population structure in the Purdue soft red winter wheat breeding population and reported the genomic regions under selection in the breeding germplasm. The majority of the loci seem to be associated with environmental adaptation, especially cold tolerance, that is indispensable for winter survival. In addition, major effects loci governing agronomic performance were identified using genome‐wide association studies. For days to heading and plant height, major loci were identified on chromosomes 7B and 6A, respectively. For test weight, number of spikes per square meter, and number of kernels per square meter, large effect loci were identified on chromosomes 1A, 4B, and 5A, respectively. This study showed population structure, signatures of recent selections, and phenotypic variations that are valuable to develop effective strategies for yield improvements.

